# Rapidly evolving marmoset MSMB genes are differently expressed in the male genital tract

**DOI:** 10.1186/1477-7827-7-96

**Published:** 2009-09-09

**Authors:** Åke Lundwall, Olivia Larne, Penelope L Nayudu, Yvonne Ceder, Camilla Valtonen-André

**Affiliations:** 1 Lund University, Department of Laboratory Medicine, Division of Clinical Chemistry, University Hospital MAS, SE-205 02 Malmö, Sweden; 2 German Primate Center, Department of Reproductive Biology, Göttingen D-37077, Germany

## Abstract

**Background:**

Beta-microseminoprotein, an abundant component in prostatic fluid, is encoded by the potential tumor suppressor gene MSMB. Some New World monkeys carry several copies of this gene, in contrast to most mammals, including humans, which have one only. Here we have investigated the background for the species difference by analyzing the chromosomal organization and expression of MSMB in the common marmoset (Callithrix jacchus).

**Methods:**

Genes were identified in the Callithrix jacchus genome database using bioinformatics and transcripts were analyzed by RT-PCR and quantified by real time PCR in the presence of SYBR green.

**Results:**

The common marmoset has five MSMB: one processed pseudogene and four functional genes. The latter encompass homologous genomic regions of 32-35 kb, containing the genes of 12-14 kb and conserved upstream and downstream regions of 14-19 kb and 3-4 kb. One gene, MSMB1, occupies the same position on the chromosome as the single human gene. On the same chromosome, but several Mb away, is another MSMB locus situated with MSMB2, MSMB3 and MSMB4 arranged in tandem. Measurements of transcripts demonstrated that all functional genes are expressed in the male genital tract, generating very high transcript levels in the prostate. The transcript levels in seminal vesicles and testis are two and four orders of magnitude lower. A single gene, MSMB3, accounts for more than 90% of MSMB transcripts in both the prostate and the seminal vesicles, whereas in the testis around half of the transcripts originate from MSMB2. These genes display rapid evolution with a skewed distribution of mutated nucleotides; in MSMB2 they affect nucleotides encoding the N-terminal Greek key domain, whereas in MSMB3 it is the C-terminal MSMB-unique domain that is affected.

**Conclusion:**

Callitrichide monkeys have four functional MSMB that are all expressed in the male genital tract, but the product from one gene, MSMB3, will predominate in seminal plasma. This gene and MSMB2, the predominating testicular gene, have accumulated mutations that affect different parts of the translation products, suggesting an ongoing molecular specialization that presumably yields functional differences in accessory sex glands and testis.

## Background

Human beta-microseminoprotein (MSMB) is synthesized from a gene located on chromosome 10, which has recently attracted much attention since genome wide association studies identified it to be connected with prostate cancer susceptibility [1,2]. It is an 11-kDa non-glycosylated protein that is expressed in many tissues, but the concentration is particularly high in prostate secretion [3]. At ejaculation MSMB is transferred with other prostate-secreted components to the seminal plasma, where it has a concentration in the range of 0.5-0.9 mg/ml in young healthy males [4]. The protein is synthesized as a precursor of 114 amino acid residues and contains a signal peptide that is removed during secretion to yield the mature protein of 94 residues, something that is also reflected in its alternative name: prostate-secreted protein of 94 amino acids (PSP_94_) [5]. Recent NMR studies show that MSMB has a unique structure, with an extended configuration, consisting of a four-stranded Greek key-motif and an exclusive domain of two two-stranded beta-sheets [6]. The only other protein that is assumed to have a similar structure is the newly identified PC3-secreted microprotein (PSMP): a protein that is highly expressed in the prostate cancer cell-line PC3 [7]. The function of MSMB is not yet known, but it forms very strong bi-molecular complexes with cysteine-rich secretory protein-3 (CRISP3) in seminal plasma and PSP94-binding protein (PSP-BP) in blood serum [8,9].

Phylogenetic studies show that MSMB is present in all this far analyzed vertebrate species and also in the chordate amphioxus [10,11]. The protein displays a very rapid evolution, as revealed by the low conservation of the primary structure between species: e.g., only 45% of the residues are identical in human and rat MSMB [12]. However, all vertebrate MSMB molecules seem to carry 10 conserved Cys that stabilize the 3D structure by forming 5 disulphide bonds [13]. In the chordate amphioxus, one of these disulfides is missing [11].

We have previously shown that some New World monkeys, e.g. the closely related cotton top tamarin (*Saguinus oedipus*) and common marmoset (*Callithrix jacchus*) of the primitive *Callithricidea *family, carry several *MSMB *in their genomes, something that is in contrast to most other vertebrate species, which carry a single *MSMB *[14]. More recently, we cloned and sequenced 5 *MSMB *from a cotton-top tamarin genomic library [15]. We concluded that 2 of them were pseudogenes, as one of them, *MSMB4*, had a deletion that shifted the reading frame and lead to premature termination, and the other, *MSMB5*, had the features of a processed pseudogene. The remaining three genes, *MSMB1*, *MSMB2*, and *MSMB3 *appeared to be functional from a structural point of view. It was not possible to investigate MSMB transcripts in tamarin tissues due to lack of material, but promoter analysis using luciferase reporter in monkey kidney COS cells showed that only *MSMB2 *displayed an activity that was comparable with that of human *MSMB*.

In this study we have extended our investigation of *MSMB *in the common marmoset in order to physically map the genes at the postulated MSMB locus and to analyze the relative expression of the genes in the male genital tract.

## Methods

### Nomenclature

Presently, the HUGO gene nomenclature committee does not provide official gene symbols to genes that are specific to non-human primates. In our earlier publications on cotton-top tamarin *MSMB*, we used gene names that were based on clone names. As these names clearly do not agree with Hugo's gene naming rules, we have decided to adopt a new nomenclature that is based on the genes' location on the chromosome. The new gene symbols are given with the old symbols written within parenthesis as follows: MSMB1 (mspA), MSMB2 (mspE), MSMB3 (mspJ), MSMB4 (mspB) and MSMB5 (mspH).

### Bioinformatics

The June 2007 *Callithrix jacchus *draft assembly, produced at the Washington University School of Medicine, St Louis, was probed with the sequence of the human *MSMB *transcript using the program BLAT, available through the University of California, Santa Cruz, Genome Bioinformatics Site [16]. DNA sequences of the housekeeping genes *GAPDH *and *CSTB *were identified by the same method using the human orthologs. The DNA contigs that were identified to contain MSMB or housekeeping gene sequences were then analyzed further using EMBOSS Tools [17] and the program package Vector NTI, which is freely available through Invitrogen's webpage [18].

### RNA isolation and cDNA synthesis

Prostate, seminal vesicles and testis from a common marmoset, kept in captivity at the German Primate Center in Göttingen, Germany, were recovered and frozen in liquid nitrogen immediately post-mortem and then stored at -80°C until further processing took place. Samples consisting of, 0.09 g prostate or 0.05 g seminal vesicle tissue were homogenized in 1.5 ml Trizol reagent (Invitrogen, Stockholm, Sweden) using a polytron homogenizer (Kinematica Inc, Lucerne, Switzerland). In the same way 0.36 g of testis tissue was homogenized in 6 ml Trizol reagent. RNA extracts were prepared according to the protocol provided with the Trizol reagent. Before cDNA synthesis, samples of 3.3 μg of total RNA were incubated for 30 min at 37°C with 1 unit of RNase-free DNase (Fermentas Sweden, Helsingborg, Sweden) in 10 μl of 10 mM Tris-HCl, pH 7.5, 2.5 mM MgCl_2 _and 0.1 mM CaCl_2_, to which 0.5 μl (20 units) of Ribolock RNase inhibitor (Fermentas) was added. To terminate the digestion, 1 μl of 25 mM EDTA was added to the samples, which subsequently were incubated for 10 min at 65°C. Each sample was then supplemented with 1 μl containing 100 pmol of oligo(dT)_18_, heated for 5 min at 65°C, cooled on ice and subjected to a collect spin. To the samples were then added 4 μl of 5 × reaction buffer (250 mM Tris-HCl, pH 8.3, 250 mM KCl, 20 mM MgCl_2 _and 50 mM DTT), 0.5 μl (20 units) of Ribolock, 2 μl of 10 mM dNTP and 1 μl (200 units) of RevetAid M-MuLV reverse transcriptase (Fermentas). Control samples were prepared in parallel by omitting the reverse transcriptase. The first strand cDNA was synthesized by incubating the samples for 1 h at 42°C. Finally, the samples were diluted with 180 μl of ultra pure water and then stored at -20°C before further analyses.

### Semi-quantitative RT-PCR

Two sets of primer pairs were synthesized for the PCR on MSMB genes. The first set had forward and reverse primers that were based on sequences of the second and the third exon and the second primer set were based on exon three and four sequences. Primers were also synthesized for two housekeeping genes: *GAPDH *and *CSTB*. The primer sequences are given in Table 1. The PCR reactions were run with 2 μl of cDNA in a total volume of 10 μl of 40 mM Tricine-KOH, 15 mM KOAc, 3.5 mM Mg(OAc)_2_, 3.75 μg/ml BSA, 0.005% Tween 20, 0.005% Nonidet-P40, 200 μM dNTP, 1 μM of forward and reverse primers, and 0.2 μl of 50 × Advantage 2 polymerase mix (Clontech, In vitro Sweden AB, Stockholm, Sweden). The PCR reactions consisted of an initial 1 min incubation at 95°C, followed by 25, 30 or 35 cycles of 30 s of denaturing at 95°C and 1 min annealing and extension at 68°C. At the end of the program there was an additional 1 min-extension at 68°C. The PCR products were analyzed by electrophoresis in 2% agarose gels that were stained with ethidium bromide (1 μg/ml). The low range MassRuler DNA ladder (Fermentas) served as molecular size marker.

**Table 1:** Nucleotide sequences of PCR primers.

**Primer name**	**Primer sequence (5' to 3' orientation)**	**Gene**	**Size (bp)**
mspAs1F	TCATGCTATTTAATACCCAATAAGATG	*MSMB1*	140
mspAs1R	ATTTCTTTTTCGAGGCAATCACATTCA	*MSMB1*	
mspEs1F	ATGGATCATGCTATGTAATACGTCATA	*MSMB2*	160
mspEs1R	AGGGAGCAACATGATATTTCTATGTCA	*MSMB2*	
mspJs1F	CATCATGCTATTTAATACTGAATGACG	*MSMB3*	149
mspJs1R	ACATGATATTTCTTTTTCTTGGCAAGT	*MSMB3*	
mspBs1F	CATCATGCTATTTAATACTGAATGACA	*MSMB4*	154
mspBs1R	GTGCAACATGATATTTCTTTTTCGCCA	*MSMB4*	
mspAs2F	AAGGTGGCAGACTGAGAACTGTGATGA	*MSMB1*	144
mspAs2R	ACCACAATATACTTGCAGTCCTCTTGC	*MSMB1*	
mspEs2F	CTGTGAGCTATGTGCTTGCCGTGACAT	*MSMB2*	189
mspEs2R	CTAGAAGCACATTACGATATCCATCCA	*MSMB2*	
mspJs2F	AAAGTGGCGGACTGACAGCTGTGACAT	*MSMB3*	152
mspJs2R	TCTTCTCCACCACAGTTAACTTGCACT	*MSMB3*	
mspBs2F	CTGACAACTGTGAGACATGTGCTTGTG	*MSMB4*	196
mspBs2R	CTAGAAGCACATTACGATATCCATCCG	*MSMB4*	
GAPDHF	AAAGTGGATGTCGTCGCCATCAATGAT	*GAPDH*	156
GAPDHR	CTGGAAGATGGTGATGGGATTTCCATT	*GAPDH*	
CTSBF	AGAAGTTCCCCGTGTTCGAGGCTGTGT	*CTSB*	126
CTSBR	GAGGGAGACTTTGGAATACTCGCAAGT	*CTSB*	

Primers belonging to primer set 1 or 2 contain the designation s1 or s2 in their name respectively. The last letter in the primer names indicate whether the oligonucleotide is priming on the coding (F) or complementary (R) strand. Size, refers to the molecular size of the expected PCR product.

### DNA sequencing

The specificity of primers was confirmed by PCR on marmoset genomic DNA followed by DNA sequencing. The PCR was run as above, but the RNA was replaced with 10 ng of genomic DNA. Material from 5 PCR reactions was pooled and purified using Jetquick (Genomed, SAVEEN Werner AB, Malmö, Sweden). The DNA concentrations were estimated following electrophoresis by comparing the staining intensity of purified PCR fragments with that of the DNA-bands in the MassRuler DNA ladder. Sequencing reactions were done with 40 ng of DNA template and 4 pmol of diluted PCR primer in a total volume of 20 μl, using the Big Dye Terminator Ready Reaction Premix diluted 1:4 and following protocols provided with supplier of the Big Dye Terminator v3.1 Cycle Sequencing Kit (Applied Biosystems, Stockholm, Sweden). The DNA sequencing was done on an ABI 3130 DNA Analyzer (Applied Biosystems) as a service by the Clinical Chemistry Department at University Hospital MAS, Malmö, Sweden.

### Quantitative RT-PCR

The cDNAs that were synthesized for semi quantitative RT-PCR were also analyzed by real-time PCR in the presence of SYBR Green. In MicroAmp Optical 384-Well Reaction Plates (Applied Biosystems), 10 μl reactions were set up by addition of 2 μl of primer mix (containing reverse and forward primers at 5 μM), 3 μl of diluted cDNA template, and 5 μl of Fast SYBR Green Master Mix (Applied Biosystems). The plate was then sealed with MicroAmp Optical Adhesive Film and real-time PCR was performed using the 7900HT Fast Real-Time PCR System (Applied Biosystems). Each primer pair was run on quadruple samples at different concentrations by serially diluting the templates between 5 and 25 times to yield at least 8 recordings for each gene in each tissue. The real-time PCR was run with the Fast SYBR Green protocol using the following cycling conditions: an initial activation step at 95°C for 20 s, followed by 40 cycles of denaturation at 95°C for 1 s and annealing and extension at 60°C for 20 s. The generated data was analyzed with the Sequence Detection System 2.3 software that is provided with the instrument. Cycle threshold (C_T_) values were calculated automatically and then slightly adjusted manually to accommodate all samples in their exponential phase.

The efficacy of the primer pairs was analyzed with prostate cDNA serially diluted 5 to 160 times. The C_T _values were plotted against the logarithms of the relative concentration and the PCR efficacy was calculated from the slope of the curve using the formula E = 10^(-1/slope)^. All primer pairs were functioning with high efficacy (Table 2).

**Table 2:** Primer pair performance.

**Primer pair**	**slope**	**R^2^**	**Relative efficacy (%)**
mspAs1F mspAs1R	-3.42	0.993	98
mspEs1F mspEs1R	-3.36	0.952	99
mspJs1F mspJs1R	-3.29	0.942	100
mspBs1F mspBs1R	-3.32	0.997	100
mspAs2F mspAs2R	-3.36	0.987	99
mspEs2F mspEs2R	-3.32	0.997	100
mspJs2F mspJs2R	-3.30	0.991	100
mspBs2F mspBs2R	-3.34	0.963	100
GAPDHF GAPDHR	-3.31	0.967	100
CTSBF CTSBR	-3.29	0.973	101

Real time PCR was done with serially diluted prostate cDNA. The recovered C_T _values were plotted against the logarithms of relative transcript concentrations and analyzed by linear regression. The PCR efficacy (E) was calculated from the equation E = 10^(-1/slope)^. A perfect doubling of PCR products in each cycle yields a slope of -3.32 and an E-value of 2.00. The relative efficacy is the observed efficacy divided by 2.

The relative concentration of different MSMB transcript in a tissue was calculated from the difference in C_T_, i.e. ΔC_T_, between the endogenous reference, which was the mean C_T _value of the primer pair generating the lowest C_T _at a given template dilution, and all measured values at this dilution. The relative transcript levels were obtained by exponentially transforming ΔC_T _values to 2^-ΔCT ^and the mean values were calculated with one standard deviation [19]. For comparison of MSMB expression in different tissues, ΔC_T _was calculated as the difference in C_T _between the mean values of *MSMB*s and the housekeeping genes *GAPDH *and *CTSB*. The sum of 2^-ΔCT ^for all four *MSMB*s in each tissue was calculated and then used to compute the ratio of *MSMB *transcripts in testis, seminal vesicles and prostate.

## Results

### Identification of 5 marmoset MSMB genes

The initial BLAT search of the common marmoset genome database identified three contigs encompassing different MSMB genes. Further analysis showed that the contig denoted 1607 carried three MSMB genes in tandem (1607:1-1607:3) and that contig 2785 also carried what appeared to be a functional gene, whereas the gene on contig 8721 had the features of a processed pseudogene. By comparing the sequences of these contigs with those of the cotton-top tamarin MSMB genes it was possible to identify the genes on contig 1607 as *MSMB4*, *MSMB3 *and *MSMB2 *and that contigs 2785 and 8721carried *MSMB1 *and *MSMB5 *respectively (Table 3). In contig 2785, we also identified a region, located 2.7 kb upstream of *MSMB1*, that is 99% similar in sequence to a piece of the *MSMB1*, encompassing exon 1, 0.6 kb upstream sequence and 2.8 kb of intron 1 sequence. This duplication is not present upstream to *MSMB1 *in the cotton top tamarin, suggesting a very recent duplication in the common marmoset.

**Table 3:** Sequence similarity between common marmoset and cotton-top tamarin MSMB genes.

**Marmoset genes**	**Tamarin genes**				
	**MSMB1**	**MSMB2**	**MSMB3**	**MSMB4**	**MSMB5**
Contig1607:1	84.1	90.6	89.6	**95.2**	89.7
Contig1607:2	84.4	84.1	**91.7**	87.5	84.4
Contig1607:3	84.4	**98.5**	85.4	90.7	96.8
Contig2785	**96.8**	84.4	85.7	84.8	83.2
Contig8721	82.9	95.9	84.4	89.9	**97.1**

The conservation of protein coding nucleotides was calculated and is expressed as percentage of conserved nucleotides. The translation initiation codon was omitted as it is not available for the processed pseudogene *MSMB5*. The bold numbers indicate highest similarity between a marmoset and a tamarin gene.

**Figure 1:**
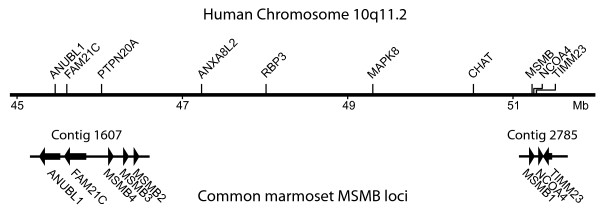
**Relative location of marmoset MSMB genes**. The upper part illustrates the approximate location of selected genes assigned to human chromosome 10q11.2. The numbers denote distance from the chromosome start point in Mb. The lower part illustrates marmoset sequence contigs with location of genes indicated by arrows or arrow heads.

By probing the human genome database with DNA sequences flanking marmoset MSMB genes, it was possible to conclude that *MSMB4*, *MSMB3*, *MSMB2 *and *MSMB1 *are all located in a genomic region that show homology with the long arm of human chromosome 10, whereas DNA flanking *MSMB5 *is homologous with the long arm of human chromosome 8. The homology search also showed that marmoset *MSMB1 *has the same position on the chromosome as the human *MSMB*, as revealed by identification of the postulated genes *NCOA4 *and *TIMM23 *at approximately the same position downstream of both the human *MSMB *and the marmoset *MSMB1*. In contrast, the genes *MSMB4*, *MSMB3 *and *MSMB2 *were assigned to a locus close to *ANUBL1*, which is situated 5.8 Mb to the centromeric side of *MSMB *in the human genome (Fig. 1). The *MSMB4 *is located 150 kb on the telomeric side of *ANUBL1 *and between them is a gene with very strong similarity to genes of the FAM21 family. In the human genome, the homologous position is occupied by *FAM21C*, but the gene in the marmoset genome is transcribed in the opposite direction to that of *FAM21C *in the human genome. The marmoset *MSMB4 *is separated from *MSMB3 *by 32 kb of intergenic DNA, and between *MSMB3 *and *MSMB2 *there is another 20 kb of intergenic DNA.

### Molecular properties of the marmoset MSMB genes and proteins

**Figure 2:**
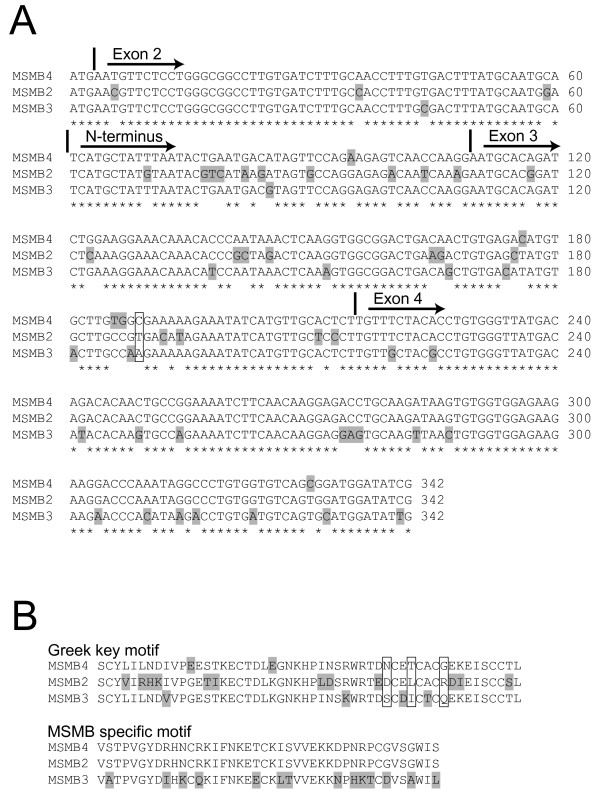
**Skewed distribution of mutations in common marmoset MSMB genes**. Aligned coding DNA (A) and amino acid (B) sequences of the three genes at the centromeric *MSMB *locus are shown. Presumed mutated residues are shaded and positions with difference in all genes are framed. Fully conserved nucleotides are indicated with stars and the location of exon/intron boundaries with vertical bars.

The four functional marmoset MSMB genes all consist of four exons separated by three introns to yield total gene sizes in the range of 12 kb to 14 kb (Table 4). All genes are flanked by conserved DNA sequences that extend 14-19 kb upstream and 3-4 kb downstream to the gene, generating regions of 32 to 35 kb that encompasses an MSMB gene with conserved flanking DNA (Table 4). The proportion of conserved nucleotides in the genes is in the range of 91-93%. The conservation of translated nucleotides is less, with a range of 85-91%, suggesting that the coding nucleotides are affected by accelerated evolution (Table 5). This is also supported by the finding that 77% of the mutated coding nucleotides generate amino acid replacements. One surprising outcome of the sequence comparisons was that the coding nucleotides of *MSMB4 *are more similar to those of both *MSMB2 *and *MSMB3 *than these two are to each other. This seems to indicate that mutations affecting *MSMB3 *and *MSMB2 *are unevenly distributed. The mutated nucleotides could be identified in the aligned sequences, which shows that most mutations affecting *MSMB3 *are located in exon 4, whereas in *MSMB2 *they are located in exon 3 and the second half of exon 2 (Fig. 2A). From this follows that mutations in *MSMB3 *affect the unique C-terminal domain of the secreted protein, whereas in *MSMB2 *they affect the N-terminal domain, with a Greek key motif (Fig. 2B). It should also be noted that only 12% of the mutations affect *MSMB4*.

**Table 4:** Sizes of marmoset MSMB genes.

**Feature**	**MSMB1**	**MSMB2**	**MSMB3**	**MSMB4**
Exon 1	35 bp	35 bp	35 bp	35 bp
Exon 2	106 bp	106 bp	106 bp	106 bp
Exon 3	106 bp	106 bp	106 bp	106 bp
Exon 4	239 bp	238 bp	237 bp	223 bp
Intron 1	7,677 bp	7,612 bp	7,028 bp	9,011 bp
Intron 2	887 bp	908 bp	897 bp	912 bp
Intron 3	5,088 bp	5,172 bp	3,618 bp	3,623 bp
Gene size	14,138 bp	14,177 bp	12,027 bp	14,016 bp
Upstream region	17 kb	16 kb	19 kb	14 kb
Downstream region	3 kb	4 kb	4 kb	4 kb
Duplicated region	34 kb	34 kb	35 kb	32 kb

Exons, introns and conserved upstream and downstream regions in marmoset genes were identified by homology with the human *MSMB *and their sizes were calculated. Duplicated region encompasses the gene and conserved flanking nucleotides.

**Table 5:** Conservation of common marmoset MSMB genes.

	**Conservation of coding nt/whole gene (%)**		
**Gene**	**MSMB2**	**MSMB3**	**MSMB4**
**MSMB1**	85/93	87/91	86/91
**MSMB2**		85/92	91/92
**MSMB3**			91/93

DNA sequences were pair wise aligned using the EMBOSS program ALIGN. The fraction conserved nucleotides were counted, omitting gapped nucleotides. The table shows the percentage of conserved coding nucleotides (nt) and conserved nucleotides in whole genes

The predicted translation products from the different MSMB genes are almost identical in molecular mass and differ by only 0.1 kDa, despite that MSMB1 has only 93 amino acid residues: one shorter than the other proteins. Their calculated isoelectric points vary from acidic for MSMB1, pI 4.9, to slightly alkaline for MSMB2, pI 8.1, with MSMB3, pI 7.2, and MSMB4, pI 6.5, located in between.

### Expression in the male genital tract

**Figure 3:**
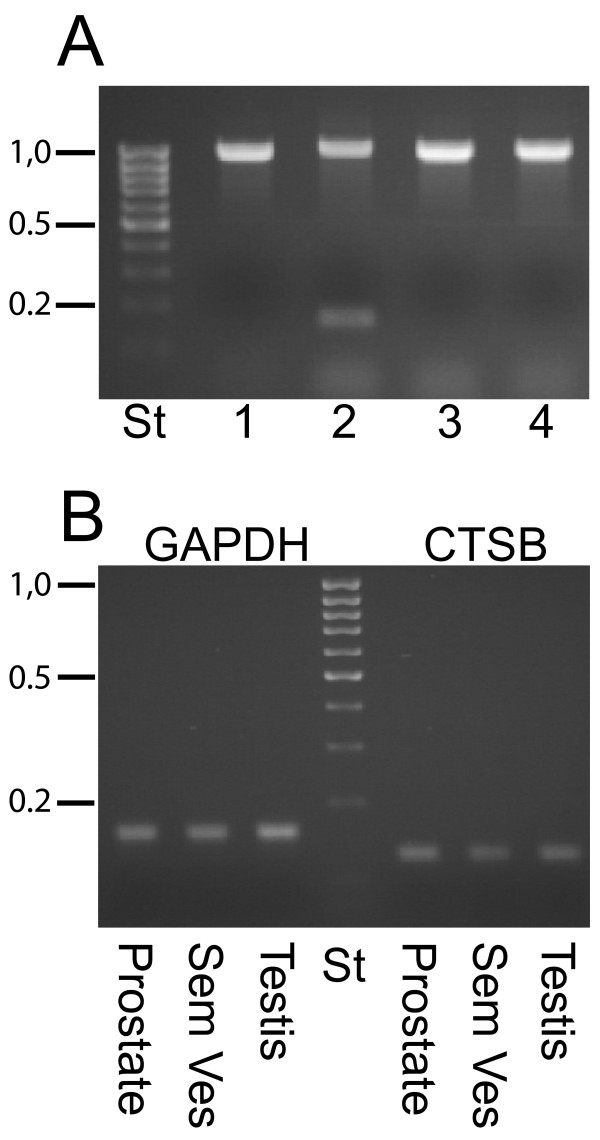
**Control PCR**. (A) The specificity of primers was tested with PCR on genomic DNA. Following 35 PCR cycles, the products were analyzed by electrophoresis in 1.2% agarose gel stained by ethidium bromide. The numbers 1, 2, 3 and 4 denote the genes *MSMB1*, *MSMB2*, *MSMB3 *and, *MSMB4 *and the label St indicates the molecular standard. (B) The relative transcript levels in different tissues were monitored in a similar way after 30 PCR cycles with the housekeeping genes *GADPH *and *CTSB*. The numbers in the left margins indicate sizes in kb of selected bands in the DNA ladder.

The relative expression of different MSMB transcripts in the genital tract of male marmoset was monitored by RT-PCR. The PCR primers were selected from DNA sequences located on different exons so that products generated from genomic DNA would also include intron and thereby differ in size from products generated from spliced mRNA. In a first control experiment, the specificity of primer pairs was tested with genomic DNA. With primers complementary to DNA sequences in exon 2 and 3 the expected PCR products should be around 1.0 kb. The PCR products generated is of the expected size, as can be seen in Fig. 3A. The specificity was also confirmed by DNA sequencing of the products. The primers used for *MSMB2 *also gave rise to a second product of 160 bp that by sequencing was identified as coming from the pseudogene *MSMB5*. The housekeeping genes *GAPDH *and *CTSB *were used as references in order to enable comparison of transcript levels in different tissues. As can be seen, the priming on the transcripts of the housekeeping genes is very specific, yielding a single PCR product with each primer pair. The similar staining intensity also indicates that the cDNA was synthesized from approximately the same quantity of mRNA (Fig. 3B).

**Figure 4:**
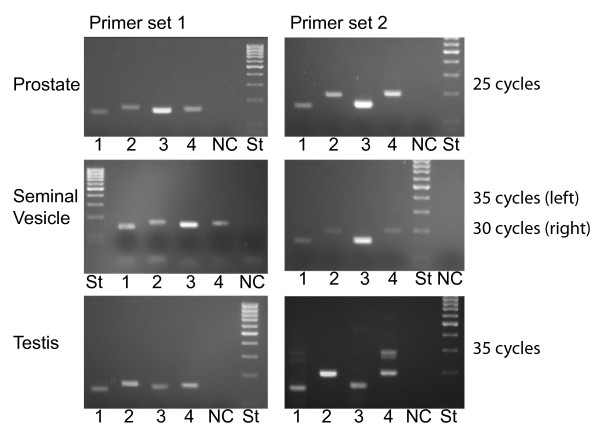
**RT-PCR**. Transcripts from prostate, seminal vesicles and testis were subjected to RT-PCR, with two different sets of primer pairs, and then analyzed by agarose gel electrophoresis. The ethidium bromide-stained gels are shown. The lettering denotes primer pair specific for *MSMB1 *(1), *MSMB2 *(2), *MSMB3 *(3), *MSMB4 *(4); negative control (NC) were run with *MSMB2 *primers on samples where reverse transcriptase were omitted during cDNA synthesis; Mass ruler low molecular size standard (St): there is 0.1 kb between bands and the molecular size of 0.5 kb is indicated by the dense band. The numbers of PCR cycles are given to the right.

The relative expression of different MSMB genes in the male genital tract was analyzed by RT-PCR with two different sets of primers. The first set, priming in exon 2 and 3, should yield products of 140 to 160 bp and the second primer set, based on sequences in exon 3 and 4, should generate products that are between 144 and 196 bp. RNA samples were treated with DNase prior to cDNA synthesis in order to overcome the potential problem with amplification of the pseudogene *MSMB5*. As can be seen, the PCR yielded products with the expected sizes for all genes in the three tissues that were analyzed, indicating that all four MSMB genes in the common marmoset are expressed (Fig. 4). In the testis, primer set 2, specific for *MSMB4*, also yielded unexpected larger PCR products that presumably represent alternatively spliced or not completely processed *MSMB4 *transcripts. In order to monitor the relative expression of genes, the number of PCR cycles had to be optimized for both primer pairs and tissues. The optimal number of PCR cycles was: in the prostate 25 for both primer pairs, in the seminal vesicles 30 or 35 depending on primer pair; and in the testis 35 for both primer pairs. The difference in number of optimal PCR cycles suggests that the level of MSMB transcripts is highest in the prostate, followed by the seminal vesicles and with testis having the lowest level. The relative staining intensity suggests that MSMB3 clearly is the dominating molecular species in both the prostate and the seminal vesicles. In the testis the transcription seems to be more evenly distributed between the different genes, but MSMB2 appears to be the dominating molecular species.

### Quantitative RT-PCR

RNA samples from marmoset prostate, seminal vesicle and testis were analyzed with real time RT-PCR in order to gather detailed information on levels of MSMB transcripts in the male genital tract. The PCR reactions were made with different template dilutions, equivalent to 2 to 10 ng of total RNA. This generated C_T_-values in the range of 13-31, where the lowest values were obtained with 10 ng of prostate RNA and the highest with 2 ng of testis RNA. The negative controls, i.e. samples without reverse transcriptase, were run with undiluted material equivalent to 50 ng of RNA. Around half of the controls yielded C_T_-values ranging from 33 to 36, but for the remainder there was no detectable signal. The difference in C_T _value between 5 times diluted samples, equivalent to 10 ng of RNA, and the matching controls was in the range of 5.8-22.6, which suggests that there is no influence of unspecific signals during the measurement of samples.

Virtually all transcripts in both the prostate and the seminal vesicles are derived from *MSMB3*, with only minor contributions from the three other *MSMB*s (Table 6). In the testis the dominating *MSMB *species is derived from *MSMB2*, which accounts for around half of the transcripts, while the other genes contribute between 9-29% (Table 6). The relative concentration of *MSMB *transcripts in the testis, prostate and seminal vesicles was estimated by comparing with transcripts of the housekeeping genes *GAPDH *and *CSTB*. The latter should have fairly constant transcript levels in all tissues and the real-time PCR yielded C_T _values of 22-23 for both of them. Using *GAPDH *for normalization yielded average ratios of 1:121:1.97 × 10^4 ^for the relative expression in testis, seminal vesicle and prostate. The same analyses with *CSTB *yielded average ratios of 1:52:1.28 × 10^4^.

**Table 6:** Relative expression of marmoset MSMB genes.

		**exon 2 and 3 primers**			**exon 3 and 4 primers**			**Average**
**Tissue**	**Gene**	**2^ΔCT^**	**σ**	**%**	**2^ΔCT^**	**σ**	**%**	**%**
Prostate	MSMB1	3.8 × 10^-3^	1.1 × 10^-3^	0.4	7.6 × 10^-3^	1.1 × 10^-3^	0.7	0.6
Prostate	MSMB2	6.9 × 10^-3^	0.5 × 10^-3^	0.7	8.5 × 10^-3^	1.9 × 10^-3^	0.8	0.8
Prostate	MSMB3	1.01	0.17	97.2	1.01	0.11	96.9	97.1
Prostate	MSMB4	1.8 × 10^-2^	0.6 × 10^-2^	1.8	1.6 × 10^-2^	0.4 × 10^-2^	1.6	1.7
Seminal Vesicle	MSMB1	4.4 × 10^-2^	0.8 × 10^-2^	4.1	7.2 × 10^-2^	0.8 × 10^-2^	6.4	5.3
Seminal Vesicle	MSMB2	1.1 × 10^-2^	0.6 × 10^-2^	1.0	1.6 × 10-^2^	0.2 × 10^-2^	1.4	1.2
Seminal Vesicle	MSMB3	1.00	0.09	93.5	1.00	0.08	89.0	91.3
Seminal Vesicle	MSMB4	1.4 × 10^-2^	0.6 × 10^-2^	1.3	3.6 × 10^-2^	0.4 × 10^-2^	3.2	2.3
Testis	MSMB1	0.34	0.19	15.1	0.62	0.19	25.8	20.5
Testis	MSMB2	0.93	0.23	41.5	1.00	0.09	41.8	41.7
Testis	MSMB3	0.25	0.09	11.2	0.15	0.07	6.2	8.7
Testis	MSMB4	0.72	0.24	32.1	0.63	0.08	26.2	29.2

Common marmoset *MSMB *were subjected to real-time RT-PCR in the presence of SYBR green. Samples were measured in quadruplicate at different RNA concentrations. Each value is based on a minimal of 8 measurements. The relative amount of transcript is given as 2^-ΔCT^, with the standard deviation σ. The fraction of *MSMB *transcripts (given as %) in a tissue was calculated by dividing individual 2^-ΔCT ^values with the sum of 2^-ΔCT ^values of the four *MSMB *in the tissue.

## Discussion

We have previously shown by Southern blotting that the common marmoset and the cotton-top tamarin have the same, or almost the same, number of *MSMB*s [14]. This is now confirmed by the demonstration of 5 *MSMB*s in the common marmoset that are orthologous with the 5 *MSMB*s in the cotton-top tamarin [15]. In addition, we identified a unique duplication in the marmoset *MSMB1*, which has created a new potential transcription initiation site around 2.7 kb upstream of the "normal" start site. Whether this new site is used for initiation of *MSMB1 *transcription or should be considered as a truncated pseudogene remains to be seen. In earlier studies on the cotton top tamarin it was not possible to determine whether the *MSMB*s, excluding the processed pseudogene *MSMB5*, are situated at a single genetic locus, but from the location of homologous regions in two of the genes it was speculated that there probably is an *MSMB *located around 20 kb downstream of *MSMB3 *[15]. In this study on the common marmoset it was found that there is indeed a gene located 20 kb downstream of *MSMB3*, but also another gene located 32 kb upstream. These three genes *MSMB2*, *MSMB3 *and *MSMB4 *constitute a unique MSMB locus that, according to the homology with human chromosome 10, is separated by several Mb from the *MSMB *locus containing *MSMB1*, which is conserved in the human and the mouse genomes. Presumably, the functional callitrichine *MSMB *have evolved by three rounds of duplication. The first presumably involved a duplication that yielded *MSMB1 *and a precursor to the genes at the unique second *MSMB *locus. We have previously shown that *MSMB3 *and *MSMB4 *are closely related [15]. It is therefore likely that a second duplication yielded *MSMB2 *and a precursor to these two genes. Finally, a third duplication yielded *MSMB3 *and *MSMB4*.

Translated exon sequences of the marmoset *MSMB*s are more dissimilar than their flanking introns, something that was previously observed also in the cotton-top tamarin [14]. Furthermore, most mutations of translated nucleotides also lead to amino acid replacements. This suggests that *MSMB*s in the callitrichine monkeys are subjected to an accelerated evolution. Analysis of mutated nucleotides in genes at the unique *MSMB *locus show that *MSMB2 *has accumulated mutations in exon 3 and the terminal half of exon 2, whereas *MSMB3 *is mostly affected in exon 4. This pattern of mutation overlaps with the domain structure of MSMB, such that in MSMB2 it is the first, Greek key, domain that is affected, whereas in MSMB3 it is the MSMB-unique second domain that is affected. The most reasonable interpretation of this phenomenon is that an evolutionary pressure has lead to specialization of the two genes. In contrast, the very few mutations detected in *MSMB4 *suggest that this gene is not under similar high evolutionary pressure. In fact, in an earlier study we demonstrated that the cotton-top tamarin *MSMB4 *is a pseudogene, something that could indicate that this gene is subjected to an ongoing purifying selection [15].

The studies on the expression show that all four functional marmoset *MSMB *are transcribed in several different cell types in the male genital tract. However, the MSMB concentration in the prostate, seminal vesicles and testis is very different, as can be seen when transcript levels are normalized with housekeeping genes. The level in the seminal vesicles is around 1% of that in the prostate and the level in the testis is even lower, by another two orders of magnitude. From this it can be concluded that almost all MSMB in seminal plasma originates from the prostate, with only minor contribution from seminal vesicles and testis. In the prostate, MSMB3 is clearly dominating, with the remaining three molecular species each contributing a few percent to the total MSMB transcript pool. A similar situation is also found in the seminal vesicles, which are dominated by MSMB3 and with only minor contribution from the other MSMB genes. In a previous investigation, we analyzed common marmoset seminal plasma with isoelectric focusing and demonstrated a predominating molecular species of MSMB with a pI value that was estimated to 7.3 [20]. This value is very close to the theoretically calculation of 7.2 for MSMB3. Thus, the high transcript level in the accessory sex glands is also reflected in a high protein concentration in seminal plasma. The isoelectric focusing also demonstrated two minor molecular species of MSMB, with pI of 6.6 and 4.9. These figures agree with the calculated pI values of 6.5 for MSMB4 and 4.9 for MSMB1, suggesting that the transcripts of these genes are also translated. In contrast, the isoelectric focusing did not display any MSMB molecule that would agree with a pI of 8.1; the calculated value of MSMB2. Whether this is due to poor translation of the *MSMB2 *transcript, instability of the translation product or other reasons, e.g. posttranslational modification, remains to be determined.

The same predominance of *MSMB3*, as seen in the accessory sex glands, was not observed in the testis. Instead it was *MSMB2 *that generated around half of the transcripts, which is interesting and could suggest that *MSMB3 *only display high transcript levels in the accessory sex glands, whereas in other organs *MSMB2 *or one of the other gene products are dominating. This is in line with the previous experiments using monkey kidney COS cells, in which only cotton-top tamarin *MSMB2 *displayed activity comparable with human *MSMB *in luciferase reporter assays [15]. Perhaps the cell and tissue specific difference in relative expression between *MSMB3 *and *MSMB2 *is mirroring the above mentioned putative specialization with accelerated evolution of either the Greek key domain or the MSMB-specific domain. This very interesting aspect could presumably be analyzed in more detail in the future, when once the function of MSMB is known.

## Conclusion

The common marmoset has orthologes of all *MSMB*, previously identified in the cotton-top tamarin, suggesting that multiple *MSMB *is a property of all Callitrichine monkeys. Transcripts of *MSMB1*, *MSMB2*, *MSMB3 *and *MSMB4 *are present in both testis and accessory sex glands, but the level in the prostate is around 100 times higher than in the seminal vesicles and 10,000 times higher than in the testis. One gene, *MSMB3*, accounts for more than 90% of the transcripts in the prostate and the seminal vesicles and yields almost all beta-microseminoprotein in seminal plasma. Marmoset MSMB displays rapid evolution, as revealed by the lower conservation of translated nucleotides compared to introns and flanking DNA. *MSMB3 *and the predominant gene in the testis, *MSMB2*, have accumulated mutations that affect different domains of beta-microseminoprotein, suggesting a specialization of these genes which might indicate different function of *MSMB *in accessory sex glands as compared to testis.

## Competing interests

The authors declare that they have no competing interests.

## Authors' contributions

ÅL designed the study, made the experiments and wrote the manuscript. OL and YC supervised the real-time PCR and took part in the analysis of data and the final design of the manuscript. PLN provided tissue samples and took part in the final design of the manuscript. CVA took part in the design of the study and the final design of the manuscript. All authors read and approved the final manuscript.
